# FEMaLe: The use of machine learning for early diagnosis of endometriosis based on patient self-reported data—Study protocol of a multicenter trial

**DOI:** 10.1371/journal.pone.0300186

**Published:** 2024-05-09

**Authors:** Dora B. Balogh, Gernot Hudelist, Dmitrijs Bļizņuks, Jayanth Raghothama, Christian M. Becker, Roman Horace, Harald Krentel, Andrew W. Horne, Nicolas Bourdel, Gabriella Marki, Carla Tomassetti, Ulrik Bak Kirk, Nandor Acs, Attila Bokor

**Affiliations:** 1 Department of Obstetrics and Gynecology, Semmelweis University, Budapest, Hungary; 2 Department of Gynecology, Center for Endometriosis, Hospital St. John of God, Vienna, Austria; 3 Rudolfinerhaus Private Clinic and Campus, Vienna, Austria; 4 Department of Computer Control and Computer Networks, Riga Technical University, Riga, Latvia; 5 Department of Biomedical Engineering and Health Systems, KTH Royal Institute of Technology, Stockholm, Sweden; 6 Oxford Endometriosis CaRe Centre, Nuffield Department of Women’s and Reproductive Health, John Radcliffe Hospital, University of Oxford, Oxford, United Kingdom; 7 Franco-European Multidisciplinary Endometriosis Institute (IFEMEndo), Clinique Tivoli-Ducos, Bordeaux, France; 8 Department of Obstetrics, Gynecology, Gynecologic Oncology and Senology, Bethesda Hospital Duisburg, Duisburg, Germany; 9 Centre for Reproductive Health, University of Edinburgh, Institute of Inflammation and Repair, Edinburgh, United Kingdom; 10 Department of Surgical Gynecology, University of Clermont Auvergne, Clermont-Ferrand, France; 11 MedEnd Institute, Budapest, Hungary; 12 Leuven University Endometriosis Center of Expertise, Leuven University Fertility Center, Department of Obstetrics and Gynecology, UZ Gasthuisberg, Leuven, Belgium; 13 Department of Public Health, Aarhus University, Aarhus, Denmark; 14 Research Unit for General Practice, Aarhus, Denmark; Public Library of Science, UNITED KINGDOM

## Abstract

**Introduction:**

Endometriosis is a chronic disease that affects up to 190 million women and those assigned female at birth and remains unresolved mainly in terms of etiology and optimal therapy. It is defined by the presence of endometrium-like tissue outside the uterine cavity and is commonly associated with chronic pelvic pain, infertility, and decreased quality of life. Despite the availability of various screening methods (e.g., biomarkers, genomic analysis, imaging techniques) intended to replace the need for invasive surgery, the time to diagnosis remains in the range of 4 to 11 years.

**Aims:**

This study aims to create a large prospective data bank using the Lucy mobile health application (Lucy app) and analyze patient profiles and structured clinical data. In addition, we will investigate the association of removed or restricted dietary components with quality of life, pain, and central pain sensitization.

**Methods:**

A baseline and a longitudinal questionnaire in the Lucy app collects real-world, self-reported information on symptoms of endometriosis, socio-demographics, mental and physical health, economic factors, nutritional, and other lifestyle factors. 5,000 women with confirmed endometriosis and 5,000 women without diagnosed endometriosis in a control group will be enrolled and followed up for one year. With this information, any connections between recorded symptoms and endometriosis will be analyzed using machine learning.

**Conclusions:**

We aim to develop a phenotypic description of women with endometriosis by linking the collected data with existing registry-based information on endometriosis diagnosis, healthcare utilization, and big data approach. This may help to achieve earlier detection of endometriosis with pelvic pain and significantly reduce the current diagnostic delay. Additionally, we may identify dietary components that worsen the quality of life and pain in women with endometriosis, upon which we can create real-world data-based nutritional recommendations.

## Introduction

Endometriosis is a chronic inflammatory disease with endometrium-like tissue outside the uterine cavity [1, 2]. It is commonly associated with chronic pelvic pain, infertility, and decreased quality of life, although asymptomatic cases exist [3–5]. The exact prevalence of endometriosis is unknown, but it may affect 5–10% of women and those assigned female at birth during their reproductive years, which equals nearly 190 million people worldwide [1]. Despite this high prevalence, recognition of the disease is inadequate, underprioritized, and research is underfunded, contributing to diagnostic delays ranging from 4 to 11 years [6–9]. Accordingly, endometriosis should be considered a public health issue, and reducing the current diagnostic delay is paramount.

Lucy is a mobile health application (Lucy app) designed to alert users with an increased risk of endometriosis to seek medical attention. Users can input their menstrual cycle information, health status, medical records or diagnoses, dietary details, lifestyle, and pain symptoms. Specialists in reproductive gynecology and health psychologists developed the app’s algorithm to continuously analyze the recorded data and alert the user when the entered symptoms are strongly associated with endometriosis.

A baseline questionnaire and a longitudinal questionnaire were developed and implemented as an additional module in the Lucy app. It collects self-reported data on symptoms of endometriosis, socio-demographics, mental and physical health, economic factors, nutrition, and other lifestyle factors [10]. By linking the collected information with existing registry-based information on diagnoses of endometriosis, healthcare use, and the big data approach, we can develop a phenotype description of individuals with endometriosis and structured clinical data [11].

So far, only one study has investigated this question with big data analysis using machine learning (ML). According to Bendifallah *et al*., endometriosis can generally be diagnosed with high diagnostic accuracy based on endometriosis-related symptoms and demographic data using ML technology [12]. The work presented by these authors was based on medical records of 800 participants of a French cohort and not on patient self-reported data.

No evidence-based advice concerning effective dietary interventions can be given to women with endometriosis [13]. Specifically, the effect of red meat, sugar, soy, dairy products, gluten, and caffeine on symptoms in women with endometriosis has not yet been systematically recorded. Therefore, we aim to investigate the effects of dietary restriction of these components on changes in quality of life, pain, and central pain sensitization. With this information, we may be able to identify nutritional components that affect the quality of life and pain in women with endometriosis. Consequently, real-world data-based dietary recommendations could be generated.

The present study aims to investigate the association between self-reported data and the clinical presentation of endometriosis. This could contribute to the classification of novel etiologic endometriosis. Such etiological findings could help to identify patterns that may categorize novel clinical subgroups of the disease. In addition, developing patient profiles may provide a novel diagnostic tool for the non-invasive, early detection of endometriosis.

### Who would benefit from the early diagnosis of the disease?

In patients with negative imaging, the definitive diagnosis of endometriosis is still laparoscopic intervention and histological analysis, which requires invasive evaluation and direct visualization of lesions, followed by histological analysis [6]. To initiate treatment, including surgery, we need a clinical hypothesis based on signs and symptoms of endometriosis, which can lead to a late diagnosis of the disease. Due to the lack of non-invasive diagnostic tools, the average delay in diagnosing endometriosis from the onset of the symptoms is 11.7 years in the United States, 8 years in the United Kingdom, and 3.9 years in Hungary [14, 15]. A non-invasive diagnostic tool would be crucial in early endometriosis diagnosis, especially the minimal-mild (#Enzian P, ASRM Stage I-II) endometriosis [16, 17].

## Objectives

### Primary objective

To establish a comprehensive real-world big data repository using the *Lucy app*. This initiative aims to identify unique clinical cohorts by leveraging various factors such as digital footprints, symptoms, patient experience, comorbidities, clinical severity, and lifestyle patterns. Using ML for big data analytics, patient profiles and structured clinical data that facilitate the earlier detection of endometriosis can be generated. Ultimately, this approach has the potential to reduce the current diagnostic delay associated with endometriosis significantly.

### Secondary objectives

- To explore whether there is an association between removing or restricting dietary components and quality of life, pain, and central sensitization.
- To investigate the connections between different types of exercise, relaxation techniques, and the symptoms or diagnosis of individuals.
- To measure the financial implications of specific diets and different dietary supplements.

## Materials and methods

### Ethics statement

This non-interventional study protocol was approved by the ethics committee of the Medical Research Council of Hungary and authorized by the National Institute of Pharmacy and Nutrition of Hungary (OGYÉI/31355/2021). It is conducted following the tenets of the Declaration of Helsinki. Before completing the questionnaires for the first time, participants will receive an informational briefing detailing the nature of the research. The application necessitates accepting the privacy policy and informational disclosure, explicitly stating that their data may be used for research purposes. The data controller only stores aggregated, anonymous data from the data provided in the *Lucy app* and the use of the application. See https://hellolucy.app/hu/protection and https://hellolucy.app/hu/agreement for detailed information.

### Study design

This study is conducted as a multicenter, parallel-group trial. Study participants are being recruited through the Lucy app. Data are collected in the following countries: Hungary, Denmark, Sweden, Germany, Austria, Italy, Romania, and Poland. Participant recruitment began on 08 February 2022 and is expected to continue until 31 December 2024.

### Eligibility criteria

5,000 women with confirmed endometriosis and 5,000 women without diagnosed endometriosis in a control group aged 18–45 years will be enrolled and followed up for one year.

### Exclusion criteria

The following exclusion criteria have been defined:

- Ongoing pregnancy.
- Lactation.
- Concern for pre-malignancy or presence/history of malignancy.

### Questionnaire

Participants will be asked to complete a baseline questionnaire once and a longitudinal questionnaire monthly for one year. The questionnaire includes questions about endometriosis symptoms, socio-demographic information, mental and physical health, known medical conditions, medication use, dietary habits, economic burden, and lifestyle factors. These include free-text responses, single or multiple-choice questions, and numerical rating scales. See Supplementary Data for the questionnaires and https://hellolucy.app/en/ for the Lucy app.

Self-reported data of the participants will be measured as follows:

- Evaluating the quality of life using the 5-level EQ-5D (EQ-5D-5L) and Endometriosis Health Profile 5 (EHP-5) [18].
- Assessing pain scores using the Visual Analogue Scale (VAS) [19].
- Examining somatic and emotional symptoms associated with central pain sensitization using the short version of the Central Sensitization Inventory (CSI-9) [20].

If using the same user account, Lucy app users can pause and resume completing the questionnaire, even on different devices. Once all questions have been answered, users can review and modify their answers on a summary screen. When they submit the questionnaire, their responses are stored on a server. At this point, the questionnaire’s content is fixed and cannot be changed.

In longitudinal studies, minimizing the drop-out rate and encouraging maximum user participation in completing the desired quantity of questionnaires is crucial. *Lucy app* users are anonymous; therefore, personal email contact is impossible. To address this challenge and reduce dropouts, the application uses several methods to notify users when the questionnaire becomes available for completion again. The questionnaire menu is prominently displayed on the application’s main page, and a pop-up window alerts users that the questionnaire is complete. Users can track the number of times and when they completed the questionnaire, but detailed completion data is not visible. The app displays a pop-up window with motivational quotes and positive messages to encourage and support users. In addition, users who complete the questionnaire are entered into a drawing as an additional incentive to participate. These measures are designed to minimize dropout and maximize user engagement in completing the questionnaires for the study.

### Data quality assessment and preprocessing

Given that real-world data inevitably contain inaccuracies, inconsistencies, text inputs, and non-qualitative information, the initial phase of our analysis is dedicated to data quality assessment and filtering. Various data quality metrics will be used, such as confirming the expected data types for each column. We will exclude records with invalid activity dates, missing activity information, users with implausible ages, and a low frequency of activity records. Statistical evaluations will be performed to assess the general data quality. For instance, the distribution of records across different days of the month should be consistent, without significant drop-offs on specific days such as the 31st. The data quality assessment metrics will understand the validity, integrity, precision, reliability, and timeliness of the data to ensure that the primary and secondary objectives can be met with the collected data. After initial filtering, we’ll dive deeper into removing non-qualitative or irrelevant data. Each symptom will be analyzed in terms of its absolute frequency and temporal distribution. Symptoms with sparse records will be discarded to prevent statistical anomalies. Any symptoms showing unrealistic temporal distributions will be subject to medical review to ensure the data’s authenticity.

### Statistical and machine learning analyses

Our dataset comprises both diagnosed and undiagnosed patients, enabling us to evaluate the quality of the data. The goal is to have a case-control ratio of 1:1 in the dataset. Specifically, records from patients diagnosed with endometriosis but not yet received treatment should highly correlate with well-known endometriosis-associated symptoms, such as pelvic pain.

The normal distribution of the data will be tested with histograms and QQ-plots. Continuous variables will be presented as averages (mean ± SD), nominal variables as numbers (n) and percentages (%). A level of statistical significance of P < 0.05 will be used. The VAS scores of the endometriosis group and the reference group will be compared with independent sample t-tests. Also, independent samples t-tests will be conducted to see whether EQ-5D-5L, CSI-9 and EHP-5 scores were different between diet-users and non-diet users. Subsequently, a one-way analysis of variance of no diet use and the various diets will be carried out for the EQ-5D-5L, CSI-9, and EHP-5 scores. A post-hoc multiple comparisons analysis (Tukey honestly significant difference) will be used to identify where the differences between these diet groups were located. Preliminary analyses will involve various correlation techniques and visual representations, including heat maps contrasting diseases against symptoms.

To explore associations between diseases, symptoms, severity, dietary habits, and lifestyle activities, we will employ Cramér’s V statistical method. Additional validation of patient segmentation will be conducted to confirm that emerging patient cohorts exhibit anticipated symptoms. Machine learning algorithms will be deployed to uncover hidden relationships or patterns. Density-Based Spatial Clustering of Applications with Noise (DBSCAN) and Uniform Manifold Approximation and Projection (UMAP) will be employed to visualize high-dimensional symptom and activity spaces in 2D or 3D representations for dimensionality reduction and clustering. Risk assessments for undiagnosed patients will be computed using Random Forest and Long Short-Term Memory (LSTM) models. The LSTM models will fully leverage the time-series data, offering predictive insights into disease outcomes based on the temporal progression of symptoms.

All the methodologies above will be tested under various scenarios, such as:

- Including all available symptoms and lifestyle/dietary activities.
- Excluding variables considered irrelevant based on existing medical literature.
- Segmenting the analysis for diagnosed versus undiagnosed patients.

These tests will help validate whether the methodologies yield consistent and reliable results. Given the substantial volume of our dataset—exceeding a million patient records—the data should be sufficient to use novel Transformer network models for pattern recognition and predictive analytics for at least low-complexity cases.

### Data safety and management

The Lucy app prioritizes the data security and anonymity of its users. It employs robust encryption measures to safeguard all data. Without the user’s password, it is technically impossible to decrypt any stored information. Furthermore, even in the unlikely event of decryption, it is essential to note that our servers do not store personal identifier information. This ensures an additional layer of protection for user privacy and data security.

The app does not ask for personal data (e.g., name, date of birth, e-mail address, etc.) from the user, and registration is not needed to use it; moreover, the IP address is not displayed and stored. The questionnaire by itself does not permit the direct identification of an individual and does not contain other information that, when combined, allows a particular individual to be indirectly identified. Accordingly, indirect identifiability is possible when data is combined with additional information that distinguishes and allows for the identification of an individual.

The free text fields within the application are securely encrypted on the servers, ensuring they remain protected. These fields are only decrypted and displayed in an unencrypted format on the user’s device. This approach allows the data to be utilized for research purposes while maintaining the utmost privacy and preventing any possibility of personal identification.

## Ethics and dissemination

All individuals must approve the privacy policy and informational disclosure, explicitly stating that their data may be used for research purposes in the *Lucy app* before participation (see Supplementary Data for Informed Consent Form). The non-interventional study protocol was approved by the ethics committee of the Medical Research Council of Hungary and authorized by the National Institute of Pharmacy and Nutrition of Hungary (OGYÉI/31355/2021) concerning scientific content and compliance with applicable research and human regulations. It is conducted following the tenets of the Declaration of Helsinki. Any modifications to the protocol will be notified and reviewed by both research ethics committees following regulations for research in the respective country. Results will be presented at national or international conferences and published in international high-impact, peer-reviewed journals.

## Conclusions

Endometriosis affects up to 190 million women worldwide and remains unresolved mainly in terms of etiology and optimal treatment strategy. It is often associated with chronic pelvic pain, infertility, and reduced quality of life. Despite this high prevalence, recognition of the disease is inadequate, underprioritized, and research is underfunded, contributing to diagnostic delays of 4 to 11 years. Therefore, the present study investigates the association between self-reported data and clinical presentation of endometriosis and aims to develop a phenotypic description of women with endometriosis. Using a big data approach, the data collected will be linked to existing registry-based information on endometriosis diagnosis and health care utilization. By unraveling the etiological aspects, we could identify patterns that may lead to categorizing novel clinical subgroups within the disease. This could enable earlier detection of endometriosis, thereby reducing current diagnostic delays. In addition, our study aims to identify different dietary components that may worsen the quality of life and pain in women with endometriosis, upon which we can make real-world data-driven nutritional recommendations. Ultimately, the findings from this multifaceted study have the potential to reduce diagnostic delays, improve patient care, and pave the way for more personalized and targeted approaches to managing endometriosis, marking a pivotal step toward enhancing the overall well-being of affected individuals.

## Supporting information

S1 Checklist SPIRIT 2013 checklist: Recommended items to address in a clinical trial protocol and related documents*. (PDF)

S1 File Baseline questionnaire and longitudinal questionnaire. (PDF)

S2 File Economic questionnaire. (PDF)

S3 File Informed consent form. (PDF)

S1 Graphical abstract (TIF)
