# Supplementary material for: FEMaLe: The use of machine learning for early diagnosis of endometriosis based on patient self-reported data—Study protocol of a multicenter trial
Source: PLoS One. 2024 May 9;19(5):e0300186. doi: 10.1371/journal.pone.0300186 (PMC11081275; doi:10.1371/journal.pone.0300186)
Supplement: S1 File — (PDF) [file pone.0300186.s002.pdf]

1. Introduction

We would like to introduce you our current study, which is part of the European collaboration of the [FEMaLe Project](https://findingendometriosis.eu/) (Finding Endometriosis using Machine Learning). The aim of our study is to find out more about patients living with endometriosis. To achieve this, we also need to ask **healthy women** who do not have endometriosis about the characteristics of their menstrual cycle, quality of life and lifestyle. In this survey, we would track your women's health indicators on a monthly basis for a year, by asking you to answer just 40-45 questions at the first time, and 23-28 questions later. The questions can be answered in a short time frame, there are no right or wrong answers, so we would encourage all participants to answer our questions honestly, according to their current menstruation cycle. The survey uses data from 12 time points to track the health of participants, the stability, or changes in the health status. These values will allow us to identify different patterns and, based on these patterns, to further improve the quality of gynaecological care according to the health conditions and needs of women today. Both the Lucy app and our research are anonymous, so neither the people running the study, nor our development engineers, nor even our staff analyzing the data will know which data comes from whom. There will be no final medical report or lab report on the results of the study. We are not able to provide feedback on individual results of participants, but we can provide feedback on aggregate results. ## Technical details of the study Name of study not involving an intervention: FEMaLe – Finding Endometriosis using Machine Learning. The investigator-in-charge: Dr. Bokor Attila PhD, egyetemi docens (Semmelweis Egyetem Szülészeti és Nőgyógyászati Klinika Baross utcai részleg, 1082 Budapest, Baross utca 27.) Questions about the app and how to use it can be asked here lucyappinfo@gmail.com ## Read more [here](https://hellolucy.app/en/female2020). The investigator-in-charge thanks you in advance for your cooperation,   Dr. Attila Bokor, MD, PhD.   Associate Professor, Semmelweis University   Faculty of Medicine, I st. Department of Obstetrics and Gynaecology

2. What is your age?
   - Numeric question

3. Where do you live?
   - Capital
   - City
   - Town
   - Village

4. What is the highest degree or level of education you have completed?
   - Less than eight elementary school classes
   - Eight elementary school classes
   - Vocational training
   - Graduation
   - College / university degree
   - Doctoral degree, PhD

5. What is your marital status?
   - Single
   - Living in a relationship
   - Married, living in a cohabiting relationship
   - Divorced
   - Widowed

6. My diagnosed disease(s):
- I have no diagnosed diseases
- Endometriosis
- Insulin resistance
- Pelvic inflammatory disease
- Myoma, fibroids
- Ovarian cyst
- Polycystic ovary syndrome (PCOS)
- Restless Leg Syndrome
- Chronic Fatigue Syndrome
- Fibromyalgia
- Temporomandibular Joint Disorder (TMJ)
- Migraine or tension headaches
- Irritable Bowel Syndrome
- Multiple Chemical Sensitivities
- Neck Injury (including whiplash)
- Anxiety or Panic Attacks
- Depression
- Other

7. If you had any disease marked in the previous question, please state the date of your diagnosis
- Free text question

8. Are you currently affected by infertility, difficulty conceiving?
- Yes
- No
- I can't tell

9. Are you sensitive to the sun?
- Yes
- No
- I can't tell

10. Number of your birthmarks:
- ~10
- ~25
- ~50
- ~100
- 100+

11. What medication are you currently receiving?
- Combined oral contraceptive
- Progesterone
- GnRH agonist
- GnRH antagonist
- Aromatase inhibitor
- Other
- I am not receiving drug therapy

12. When was the first day of your last period?
- Free text question

13. Are you currently pregnant?
- Yes
- No

14. Please describe your **lifestyle (even your diet, exercise, sleep)** with a few individual words
- Multiple free text question

15. Please describe your **health** with a few individual words
- Multiple free text question

16. Please describe **yourself** with a few individual words
- Multiple free text question

17. Has there been any change in your pain symptoms since you last completed the questionnaire?
- No
- Yes

18. Please mark on the scale below how strong you have experienced YOUR PAIN in your current or recent cycle **on the bleeding days (dysmenorrhea)**

Min: 0 - Max: 10

19. Please mark on the scale below how strong you have experienced YOUR PAIN in your current or recent cycle **during nonbleeding day (chronic pelvic pain)**

Min: 0 - Max: 10

20. Please mark on the scale below how strong you have experienced YOUR PAIN in your current or recent cycle **during or after sexual intercourses (dyspareunia)**

Min: 0 - Max: 10

21. Please mark on the scale below how strong you have experienced YOUR PAIN in your current or recent cycle **during defecation (dyschezia)**

Min: 0 - Max: 10

22. Please mark on the scale below how strong you have experienced YOUR PAIN in your current or recent cycle **during urination (dysuria)**

Min: 0 - Max: 10

23. Your health today.

Under the next 5 questions, please tick the box that best describes your health **TODAY**.

24. **MOBILITY**
- I have no problems in walking about
- I have slight problems in walking about
- I have moderate problems in walking about
- I have severe problems in walking about
- I am unable to walk about

25. **SELF-CARE**
    - I have no problems washing or dressing myself
    - I have slight problems washing or dressing myself
    - I have moderate problems washing or dressing myself
    - I have severe problems washing or dressing myself
    - I am unable to wash or dress myself

26. **USUAL ACTIVITIES** (e.g. work, study, housework, family or leisure activities)
    - I have no problems doing my usual activities
    - I have slight problems doing my usual activities
    - I have moderate problems doing my usual activities
    - I have severe problems doing my usual activities
    - I am unable to do my usual activities

27. **PAIN/DISCOMFORT**
    - I have no pain or discomfort
    - I have slight pain or discomfort
    - I have moderate pain or discomfort
    - I have severe pain or discomfort
    - I have extreme pain or discomfort

28. **ANXIETY/DEPRESSION**
    - I am not anxious or depressed
    - I am slightly anxious or depressed
    - I am moderately anxious or depressed
    - I am severely anxious or depressed
    - I am extremely anxious or depressed

29. Please mark on the scale to indicate how your health is TODAY.
    Min: 0 - Max: 100

30. Do you have a diagnosed case of **endometriosis**?
    - No, I don't
    - Yes, I do

31. What type of endometriosis were you diagnosed with on your final report?
    - I.
    - II.
    - III.
    - IV.
    - I can't tell

32. Has there been a qualitative change in your quality of life related to endometriosis since you last completed the questionnaire?
    - No
    - Yes

33. During the last 4 weeks, how often, because of your endometriosis **have you found it difficult to walk because of the pain**?
    - Never
    - Rarely
    - Sometimes
    - Often
    - Always

34. During the last 4 weeks, how often, because of your endometriosis **have you felt as though your symptoms are ruling your life**?
- Never
- Rarely
- Sometimes
- Often
- Always

35. During the last 4 weeks, how often, because of your endometriosis **have you had mood swings**?
- Never
- Rarely
- Sometimes
- Often
- Always

36. During the last 4 weeks, how often, because of your endometriosis **have you felt others do not understand what you are going through**?
- Never
- Rarely
- Sometimes
- Often
- Always

37. During the last 4 weeks, how often, because of your endometriosis **have you felt your appearance has been affected**?
- Never
- Rarely
- Sometimes
- Often
- Always

38. Being tired - Please pick the best response for the following statements
- Never
- Rarely
- Sometimes
- Often
- Always

39. My muscles feel stiff and achy.
- Never
- Rarely
- Sometimes
- Often
- Always

40. I feel pain all over my body.
- Never
- Rarely
- Sometimes
- Often
- Always

41. I have headaches.
   - Never
   - Rarely
   - Sometimes
   - Often
   - Always

42. I do not sleep well.
   - Never
   - Rarely
   - Sometimes
   - Often
   - Always

43. I have difficulty concentrating.
   - Never
   - Rarely
   - Sometimes
   - Often
   - Always

44. Stress makes my physical symptoms get worse.
   - Never
   - Rarely
   - Sometimes
   - Often
   - Always

45. I have muscle tension in my neck and shoulders.
   - Never
   - Rarely
   - Sometimes
   - Often
   - Always

46. I have difficulty remembering things.
   - Never
   - Rarely
   - Sometimes
   - Often
   - Always

47. Mark which foods and nutrients you have **completely removed** from your nutrition in the last month
   - Sugar
   - Gluten
   - Coffee
   - Soy
   - Dairy products
   - Red meat

48. Mark which foods and nutrients you have **limited** in your diet in the past month
- Sugar
- Gluten
- Coffee
- Soy
- Dairy products
- Red meat

49. Do you agree that your data entered in Lucy can be analyzed together with your completed questionnaire to help us obtain more detailed and accurate research results?
- Yes
- No
