# Supplementary material for: FEMaLe: The use of machine learning for early diagnosis of endometriosis based on patient self-reported data—Study protocol of a multicenter trial
Source: PLoS One. 2024 May 9;19(5):e0300186. doi: 10.1371/journal.pone.0300186 (PMC11081275; doi:10.1371/journal.pone.0300186)
Supplement: S2 File — (PDF) [file pone.0300186.s003.pdf]

1. Highest level of education:
a. primary school
b. secondary school (school leaving certificate / GCSE / O levels)
c. further education/college (A levels / vocational qualifications)
d. apprenticeship
e. higher education (university)
f. postgraduate

2. Are you currently taking any dietary supplements?
a. vitamins (D, C, E)
b. curcumin
c. magnesium
d. trace elements (zinc, iodine, selenium)
e. NAC (N-acetyl-L-cysteine)
f. fish oil (omega-3)
g. resveratrol
h. medicinal herbs (ginger, milk thistle, evening primrose, chaste berry, etc.)
i. prebiotics
j. other
k. not taking

3. If you take them, how long have you been taking them?
a. less than 3 months
b. 3-6 months
c. 7-9 months
d. 10-12 months
e. more than 1 year

4. On average, how much do you spend on food supplements per month?
a. less than €5
b. €5-15
c. €15-25
d. €25-35
e. more than €35
f. don't know

5. Are you on a special diet?
a. gluten-free
b. dairy-free
c. sugar-free
d. low FODMAP
e. vegan
f. macrobiotic
g. organic
h. other
i. not considered

6. If you are on a diet, how long have you been on it?
a. less than 3 months
c. 3-6 months
d. 6-12 months
e. 1-2 years
f. more than 2 years

7. What are you looking for when buying special foods (e.g. gluten-free food, plant-based milk)?
a. price
b. brand

8. On average, how much does your household spend on food per month?
a. less than €200
b. €200-250
c. €250-300
d. €300-400
e. more than €400

9. How often did you visit your doctor per year before starting your diet/supplementation?
a. less than 1 time
b. 1 time
c. 2 times
d. 3-4 times
e. more than 4 times

10. How often do you see a doctor each year since you started taking the diet/dietary supplement?
a. less than 1 time
b. 1 time
c. 2 times
d. 3-4 times
e. more than 4 times

11. Do you use public or private gynecological care?
a. public
b. private
c. both

12. Have you ever consulted a dietician?
a. yes, once
b. a few times
c. regularly
d. no

13. Do you use public or private dietetic advice?
a. public
b. private
c. both

14. How many people live in your household?
Free text question

15. How much is the net income of your household?
a. less than €25,000/year
b. €25,000 - 35,000/year
c. €35,000 - 50,000/year
d. €50,000 - 70,000/year
e. more than €70,000/year
f. prefer not to answer

16. Is there any other circumstance that you feel is important to mention concerning food?
Free text question
