# Supplementary material for: FEMaLe: The use of machine learning for early diagnosis of endometriosis based on patient self-reported data—Study protocol of a multicenter trial
Source: PLoS One. 2024 May 9;19(5):e0300186. doi: 10.1371/journal.pone.0300186 (PMC11081275; doi:10.1371/journal.pone.0300186)
Supplement: S3 File — (PDF) [file pone.0300186.s004.pdf]

We would like to introduce you our current study, which is part of the European collaboration of the [FEMaLe Project](https://findingendometriosis.eu/) (Finding Endometriosis using Machine Learning). The aim of our study is to find out more about patients living with endometriosis. To achieve this, we also need to ask **healthy women** who do not have endometriosis about the characteristics of their menstrual cycle, quality of life and lifestyle.

In this survey, we would track your women's health indicators on a monthly basis for a year, by asking you to answer just 40-45 questions at the first time, and 23-28 questions later. The questions can be answered in a short time frame, there are no right or wrong answers, so we would encourage all participants to answer our questions honestly, according to their current menstruation cycle.

The survey uses data from 12 time points to track the health of participants, the stability, or changes in the health status. These values will allow us to identify different patterns and, based on these patterns, to further improve the quality of gynaecological care according to the health conditions and needs of women today.

Both the Lucy app and our research are anonymous, so neither the people running the study, nor our development engineers, nor even our staff analyzing the data will know which data comes from whom. There will be no final medical report or lab report on the results of the study. We are not able to provide feedback on individual results of participants, but we can provide feedback on aggregate results.

Technical details of the study

Name of study not involving an intervention: FEMaLe – Finding Endometriosis using Machine Learning.

The investigator-in-charge: Dr. Bokor Attila PhD, egyetemi docens (Semmelweis Egyetem Szülészeti és Nőgyógyászati Klinika Baross utcai részleg, 1082 Budapest, Baross utca 27.)

Questions about the app and how to use it can be asked here lucyappinfo@gmail.com

Read more https://hellolucy.app/en/female2020

The investigator-in-charge thanks you in advance for your cooperation,
Dr. Attila Bokor, MD, PhD.
Associate Professor, Semmelweis University
Faculty of Medicine, I st. Department of Obstetrics and Gynaecology
