# Supplementary figures and images for: FEMaLe: The use of machine learning for early diagnosis of endometriosis based on patient self-reported data—Study protocol of a multicenter trial
Source: PLoS One. 2024 May 9;19(5):e0300186. doi: 10.1371/journal.pone.0300186 (PMC11081275; doi:10.1371/journal.pone.0300186)

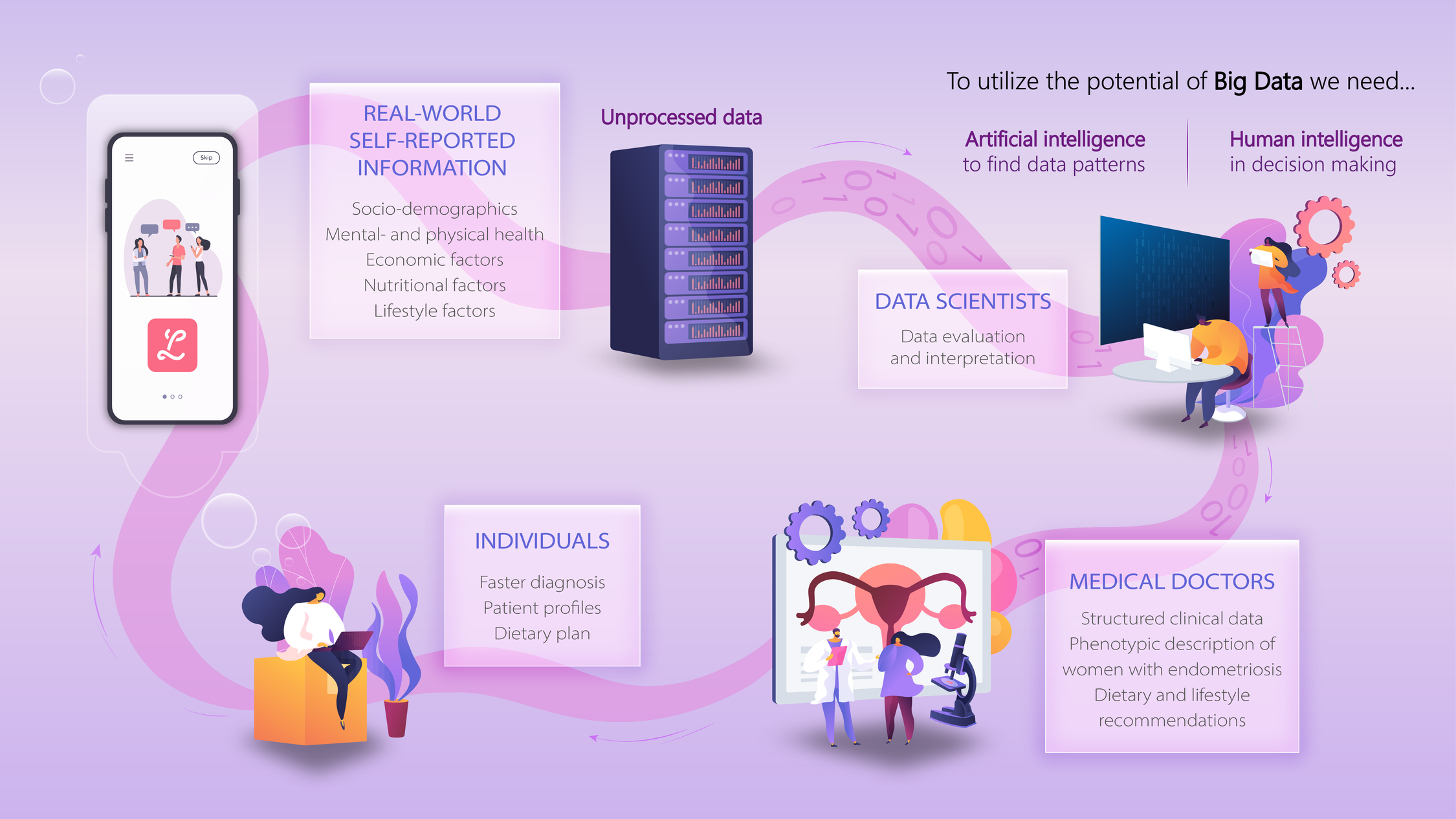

Supplement: S1 Graphical abstract — (TIF) [file pone.0300186.s005.tif]
